# Complete Genome Sequence of Psittacine Adenovirus 1, Identified from Poicephalus senegalus in Italy

**DOI:** 10.1128/MRA.01037-18

**Published:** 2018-09-20

**Authors:** Adelaide Milani, Gianpiero Zamperin, Alice Fusaro, Annalisa Salviato, Luca Bano, Luca Zandonà, Romina Brunetta, Isabella Monne

**Affiliations:** a Division of Comparative Biomedical Sciences, Istituto Zooprofilattico Sperimentale delle Venezie, Legnaro (Padua), Italy; b SCT2, Istituto Zooprofilattico Sperimentale delle Venezie, Legnaro (Padua), Italy; Louisiana State University

## Abstract

Using a metagenomics approach, we were able to determine for the first time the full-genome sequence of a psittacine adenovirus 1 isolate that was recovered from the liver of a dead Senegal parrot (Poicephalus senegalus) in Italy. The results of the phylogenetic investigations revealed the existence of high genetic diversity among adenoviruses circulating in psittacine birds.

## ANNOUNCEMENT

Psittacine birds have been shown to be infected by members of the Atadenovirus (1) and Aviadenovirus (2, 3) genera, and these infections have been associated with a wide range of signs, such as diarrhea, anorexia, and depression. Until now, only 2 full-genome sequences of adenoviruses infecting psittacine birds have been available in public databases (GenBank accession numbers KJ675568 and KX577802).

The Poicephalus senegalus bird analyzed in this work died within 24 h from the onset of depression and anorexia. Gross lesions included an enlarged, mottled, and friable liver with necrotic foci. The proventriculus was distended, and the intestine showed a serum-hemorrhagic enteritis. Examination by transmission electron microscopy and L1 hexon PCR on the intestine, proventriculus, and liver confirmed the presence of adenovirus in the liver. Total DNA from the liver was extracted using the High Pure PCR template preparation kit (Roche, Mannheim, Germany), according to the manufacturer’s instructions, investigated by a next-generation sequencing (NGS) approach using the Nextera XT DNA sample preparation kit, and processed on an Illumina MiSeq instrument with MiSeq reagent kit V3 (2 × 300-bp paired-end [PE] mode; Illumina, San Diego, CA, USA).

Sequencing yielded 14,215,807 paired-end reads 301 bp long, which were quality filtered and taxonomically classified by (i) aligning against the integrated NT database (version 12, February 2018) using BLAST 2.7.1+ (4), with default parameters, and against the integrated NR database (version 12, February 2018) using DIAMOND version 0.9.17 (5), with default parameters; (ii) filtering out alignment hits with E values larger than 1 × 10^−3^; and (iii) feeding remaining alignment hits to MEGAN ue version 6.10.8 (6). Reads taxonomically classified as belonging to the *Adenoviridae* family were selected and *de novo* assembled using IDBA-UD version 1.1.1 (7), using default parameters and the multi-*k*-mer approach (minimum value, 24; maximum value, 124; increment, 10). Only a single contig with a length comparable to the size of the *Adenoviridae* genome was obtained. All reads belonging to the *Adenoviridae* family were subsequently aligned against the longest contig obtained from the *de novo* assembly using BWA version 0.7.12 (8) with standard parameters. The alignment was manually revised with Tablet (9) to make sure that all nucleotides were the consensus ones and no reads were misaligned, as well as to avoid the risk of misassembly.

The final genome obtained resulted in a consensus sequence 38,694 nucleotides long, showing a sequence coverage of 41,957-fold and 51.3% G+C content. A comparison with full Adenovirus genomes available in GenBank (June 2018) revealed 90% query cover and 84% identity with psittacine adenovirus B (PsAdV-B; GenBank accession number KX577802). Fifty-five open reading frames (ORFs) longer than 50 amino acids were predicted within the genome using the ORFfinder tool (https://www.ncbi.nlm.nih.gov/orffinder/); 31 ORFs showed an amino acid similarity with PsAdV-B ranging from 46% to 97% (86% on average). BLAST results of the L1 hexon region showed 83% and 99% nucleotide identities with PsAdV-B (GenBank accession number KX577802) and PsAdV-1 (GenBank accession number EF442329), respectively; however, only a partial hexon sequence (L1) is available for PsAdV-1.

Phylogenetic analysis of the nucleotide and amino acid sequences of the L1 hexon (3, 10) was performed with the maximum likelihood (ML) method using the PhyML 3.1 software (11). Classification of aviadenovirus genotypes based on phylogenetic trees indicates that the virus under study belongs to the PsAdV-1 genotype, previously identified in a Psittacula alexandri bird (12).

The complete characterization of the full-genome sequence of psittacine adenovirus 1 obtained from a Poicephalus senegalus bird was made possible for the first time thanks to our study, which also suggested the likely existence of high genetic diversity among adenoviruses circulating in psittacine birds.

### Data availability.

MiSeq raw data were submitted to the NCBI Sequence Read Archive (SRA) under accession number SRR7426217. The complete PsAdV-1 genome sequence has been deposited in GenBank under accession number MH580295.
